# Relationship of MMP-14 and TIMP-3 Expression with Macrophage Activation and Human Atherosclerotic Plaque Vulnerability

**DOI:** 10.1155/2014/276457

**Published:** 2014-08-24

**Authors:** Jason L. Johnson, Nicholas P. Jenkins, Wei-Chun Huang, Karina Di Gregoli, Graciela B. Sala-Newby, Vincent P. W. Scholtes, Frans L. Moll, Gerard Pasterkamp, Andrew C. Newby

**Affiliations:** ^1^Laboratory of Cardiovascular Pathology, School of Clinical Sciences, Level 7, Bristol Royal Infirmary, Bristol BS2 8HW, UK; ^2^School of Clinical Sciences, University of Bristol, Bristol BS2 8HW, UK; ^3^University Medical Centre, 3584 Utrecht, The Netherlands

## Abstract

Matrix metalloproteinase-14 (MMP-14) promotes vulnerable plaque morphology in mice, whereas tissue inhibitor of metalloproteinases-3 (TIMP-3) overexpression is protective. MMP-14^hi^  TIMP-3^lo^ rabbit foam cells are more invasive and more prone to apoptosis than MMP-14^lo^  TIMP-3^hi^ cells. We investigated the implications of these findings for human atherosclerosis.* In vitro* generated macrophages and foam-cell macrophages, together with atherosclerotic plaques characterised as unstable or stable, were examined for expression of MMP-14, TIMP-3, and inflammatory markers. Proinflammatory stimuli increased MMP-14 and decreased TIMP-3 mRNA and protein expression in human macrophages. However, conversion to foam-cells with oxidized LDL increased MMP-14 and decreased TIMP-3 protein, independently of inflammatory mediators and partly through posttranscriptional mechanisms. Within atherosclerotic plaques, MMP-14 was prominent in foam-cells with either pro- or anti-inflammatory macrophage markers, whereas TIMP-3 was present in less foamy macrophages and colocalised with CD206. MMP-14 positive macrophages were more abundant whereas TIMP-3 positive macrophages were less abundant in plaques histologically designated as rupture prone. We conclude that foam-cells characterised by high MMP-14 and low TIMP-3 expression are prevalent in rupture-prone atherosclerotic plaques, independent of pro- or anti-inflammatory activation. Therefore reducing MMP-14 activity and increasing that of TIMP-3 could be valid therapeutic approaches to reduce plaque rupture and myocardial infarction.

## 1. Introduction

Plaque rupture accounts for three quarters of myocardial infarctions [1]. Weakening of the fibrous cap owing to net degradation of extracellular matrix is believed to play an important part in plaque rupture [2]. Foam-cell macrophages (FCMs) are an abundant source of several matrix metalloproteinases (MMPs) in human plaques [3, 4]. Protective effects of certain MMPs on smooth muscle migration and proliferation have been identified from experimental studies but high levels of MMP production, especially from FCMs, have been implicated in plaque progression and rupture [2]. Attention has focused recently on MMP-14, which is an activated membrane type MMP, and a tissue inhibitor of MMPs (TIMP-3), which binds to the extracellular matrix. Given these properties, MMP-14 and TIMP-3 are likely to play important divergent roles in the regulation of macrophage pericellular proteolysis. Adoptive transfer of leukocytes lacking MMP-14 into LDL-receptor null mice increased plaque collagen content, implying a harmful collagen-depleting role of MMP-14 in plaque stability [5]. Conversely, transgenic overexpression of TIMP-3 in mouse macrophages reduced atherosclerosis formation and improved markers of plaque stability [6]. A MMP-14^hi^  TIMP-3^lo^ subpopulation of rabbit FCMs was shown to have greater proteolytic capacity, ability to invade through synthetic extracellular matrix (ECM), and greater propensity to undergo apoptosis, all of which would be expected to favour plaque rupture [7]. MMP-14^hi^  TIMP-3^lo^ FCMs were identified in human atherosclerotic plaques [7] but the mechanisms responsible for generating the MMP-14^hi^  TIMP-3^lo^ FCMs and their relationship to plaque vulnerability in man were not previously investigated.

Macrophages can express a spectrum of different activation states dependent on pro- and anti-inflammatory signals [8, 9], although the effects on MMP-14 and TIMP-3 expression are not fully documented. For example, activation by Toll-like receptor agonists [10] and other inflammatory cytokines acting through nuclear factor-*κ*B (NF-*κ*B) upregulates several MMPs [11]. Such activated macrophages have been associated with unstable plaque morphology, whereas deactivated or alternatively activated macrophages are associated with increased plaque stability in most [12–14], although not all, studies [15]. Phenotypes resulting from oxidised phospholipids [16] or by the action of haem at sites of thrombosis and intraplaque haemorrhage have also been recently distinguished [17–19].

To clarify the nature and role of MMP-14^hi^  TIMP-3^lo^ FCMs we investigated their activation* in vitro* with pro- and anti-inflammatory molecules. We then investigated whether macrophage populations with opposing expression of MMP-14 and TIMP-3 colocalise with markers of macrophage activation and correlate with histological markers of vulnerability to rupture in human carotid atherosclerotic plaques.

## 2. Materials and Methods

### 2.1. Carotid Artery Specimens

Carotid endarterectomy specimens came from the AtheroExpress Biobank, the details of which have been described in detail elsewhere [20–22]. Briefly, patient demographics including cardiovascular risk factors, previous medication use, and presenting symptoms were recorded. Atherosclerotic plaques were dissected by a dedicated technician into 5 *μ*m thick cross-sectional segments. Histological staining (haematoxylin and eosin (HE), Picrosirius Red (PS)) were performed on the so-called culprit lesion (segment with largest plaque area). The area occupied by following parameters was scored semiquantitatively as described previously [20–22]: atheroma core (HE, PS), calcification, (HE), collagen (PS), smooth muscle cells (*α*SM-ctin; M0851; Dako), and macrophages (CD68; M0814; Dako) (Table 1). Smooth muscle cells and macrophages were also scored quantitatively by using a microscope equipped with a digital camera and using AnalySIS 3.2 software (Soft Imaging System GmbH, Munster, Germany). The amount of microvessels was quantified by using an anti-CD34 antibody; details have been described previously [20–22]. As specified in the AtheroExpress protocol, histological sections were classified according to overall appearance into: “atheromatous lesions” containing a large lipid core (>40% of plaque area), high macrophage infiltration with low smooth muscle cell and collagen content, “fibrous lesions” with a small (<40%) or absent lipid core, low macrophage content and high smooth muscle cell and collagen content, and “fibrous-atheromatous lesions” as an intermediate between the two other phenotypes. The inter- and intraobserver variability for this classification has been described previously [23]. Adjacent sections were then taken for the histological analyses reported here, which were performed and analysed by 2 additional operators who were blinded as to the lesion classification.

### 2.2. Immunohistochemistry

Sections were dewaxed in Clearene and rehydrated in absolute alcohol. After rinsing in distilled water, slides were subjected to heat-induced antigen retrieval by incubation in citrate buffer (10 mM citric acid in 1x PBS, pH 6.0) and microwaved for 10 minutes at full power. Slides were allowed to cool for 30 minutes and then washed with PBS (3 × 2 minutes). For brightfield or fluorescence IHC, 50 *μ*L of 1% bovine serum albumin or Image-iT FX Signal Enhancer (Invitrogen, Life Technologies, Paisley, UK), respectively, was placed on sections and incubated at room temperature for 30 minutes. The solution was then removed by tapping the slides and 50 *μ*L of the applicable primary antibody (as detailed in Table 1) added to the samples and incubated either overnight at 4°C or for 1 hour at room temperature. Sections were then washed in PBS (3 × 2 minutes each) and incubated in the desired secondary antibody as detailed in Table 1. For fluorescent labelling, sections were incubated in the dark for 1 hour at room temperature. Sections were then washed in PBS (3 × 2 minutes) and mounted with ProLong Gold antifade reagent containing DAPI (Invitrogen, Life Technologies, Paisley, UK) to identify the nuclei. A negative control where the primary antibody was replaced with the relevant species IgG at the same dilution was always included and the cells were counted in 6 × 20 magnification fields. Positive cells were counted and expressed as a percentage of total nucleated cells. The percentage of CD68 positive macrophages that were also positively stained for other proteins of interest was counted by comparing serial sections or after dual-immunohistochemistry if the antibodies were suitable, by independent observers (JLJ, NPJ, VWPS) and the results averaged, as described previously [24].

For dual fluorescence, primary antibodies from different species were used and incubated together. Subsequently, species-specific fluorophore conjugated secondary antibodies were used to yield either a red fluorescent product at the site of the antigen (AlexaFluor 594) or a green fluorescent product at the site of the antigen (AlexaFluor 488). Sections were then mounted in ProLong Gold Antifade Reagent with 4′,6-diamidino-2-phenylindole (Life Technologies, Paisley, UK; P-36931) to fluorescently label nuclei blue. The specificity of the immunolabeling was demonstrated by inclusion of a negative control using isotype-specific nonimmune serum or IgG. Images were acquired from a fluorescence microscope using ImageProPlus image analysis software (Media Cybernetics) and merged if required before further analysis. The percentage of MMP-14 positive macrophages that stained for other proteins was counted within each lesion and expressed as percentage of total MMP-14 positive macrophages.

### 2.3. Studies with Isolated Primary Human Macrophages

Peripheral blood mononuclear cells were isolated by differential centrifugation from whole blood of healthy donors, which were collected under South West 4 Research Ethics Committee reference 09/H0107/22. Blood (24 mL per donor) was diluted with Dulbecco's Phosphate Buffered Saline (PBS) without calcium and magnesium (Lonza) 1x (ratio 1 : 1). The diluted samples were subjected to density gradient separation on Ficoll Paque Plus (ratio 1 : 1) (GE Healthcare Life Sciences, Buckinghamshire, UK) and centrifuged. After centrifugation the PBMC layer was collected and washed in Hank's Balanced Salt Sodium (HBSS) with phenol red without calcium and magnesium (Lonza). Monocytes were isolated by adhering the peripheral blood mononuclear cells to tissue culture plastic for 2 hours at a concentration of 2.5 × 10^6^ cells/mL. Monocytes were cultured in RPMI media with 2 mM L-glutamine, 100 IU/mL penicillin, 100 *μ*g/mL streptomycin, 10% fetal bovine serum (FBS; Lonza, Sigma), and 20 ng/mL recombinant human macrophage-colony-stimulating factor (R & D systems). The medium included 85 *μ*g/mL of fully oxidised low-density lipoprotein, oxLDL (Intracel) from day 5 when foam-cell macrophages (FCM) were generated. At Day 7, the medium was removed and replaced with RPMI media supplemented with 5% FCS. Recombinant human interferon-*γ* (10 ng/mL R & D systems) and lipopolysaccharide-LPS (200 ng/mL, Escherichia coli 026:B6, Sigma-Aldrich) were added for 24 hours to generate classically activated macrophages (M1) [25] or recombinant human interleukin-4 (20 ng/mL R & D systems) to obtain alternatively activated macrophages (M2) [26].

### 2.4. Reverse Transcription-Polymerase Chain Reaction (RT-PCR)

RNA samples from macrophages were collected using RLT solution (Qiagen) with *β*-mercaptoethanol (*β*-ME; 1 : 100 dilution). Total RNA was extracted using RNeasy Mini kit (Qiagen) according to the manufacturer's instructions. RNA was quantified using a Nanodrop Spectrophotometer and cDNA generated using a QuantiTect Reverse Transcription Kit (100–200 ng RNA per reaction Qiagen). Real-time quantitative PCR was performed in a Roche Light Cycler 1.5 to quantify the steady-state concentration of RNA using a QuantiTect SYBR Green PCR Kit and primers as detailed in Table 2. The reaction contained 3.6–7.3 ng RNA and 0.5 *μ*M primers. Denaturation for 15 min at 95°C was followed by 60 cycles of denaturation (15 seconds at 95°C), annealing for 20 seconds, and extension for 25 seconds at 72°C. Copy numbers of mRNA were calculated using standard curves constructed using the respective PCR products eluted from agarose gels. All fragments were sequenced to confirm identity (Cogenics, Takeley, UK). Primers used and their annealing temperatures are in Table 2.

### 2.5. Western Blotting

Adherent cells were washed with PBS on ice and then lysed with ice-cold 1% SDS lysis buffer. The Bicinchoninic acid (BCA) Kit (Pierce) was used to estimate protein concentration (all in duplicate) of cell lysates according to kit protocol. Protein concentration was read at 560 nm on a Multiskan Ascent (Thermo Electron Corporation) plate reader. NuPAGE Novex Bis-Tris Mini Gel system (Invitrogen, Life Technologies, Paisley, UK) was used for western blot experiments. 10 *μ*g of total protein was loaded on 4–12% Bis-Tris-HCl buffered (pH 6.4) polyacrylamide precast gels (Invitrogen, Life Technologies, Paisley, UK) and run in MES-SDS Running Buffer 1x (Invitrogen, Life Technologies, Paisley, UK) according to manufacturer instructions. After electrophoresis, gels were transferred to a 0.45 *μ*m nitrocellulose membrane in NOVEX NuPAGE Transfer Buffer 1x plus 5% Methanol (v/v). Following transfer, the nitrocellulose membrane was blocked in 5% milk solution in Tris buffered solution containing Tween (TBS-T) (200 mM Tris, 2% Tween 20, pH 7.6). The membrane was then incubated over night at 4°C using the antibodies described in Table 1 diluted in 2 mL of SignalBoost Solution 1 (Calbiochem-Novabiochem Ltd., Nottingham, UK). The following day the membrane was incubated with 5% milk solution in TBS-T and then with the applicable horseradish peroxidase (HPR)-labelled secondary antibody (Dako, Dorset, UK) diluted 1 : 1000 in SignalBoost Solution 2 (Calbiochem-Novabiochem Ltd., Nottingham, UK). After incubation the membrane was washed in TBS-T. To detect the peroxidise labelled proteins, an enhanced chemiluminescence (ECL) detection system (GE Healthcare Life Sciences, Buckinghamshire, UK) was used. The membrane was incubated with ECL reagent mix (solution A and solution B at a ratio of 1 : 1) and exposed to X-ray film for the desired length of time. As a loading control 0.5 *μ*g/mL of either anti-*β*-actin antibody (A2228, Sigma) or anti-GAPDH antibody (MAB374, Millipore) was used. Detected bands were quantified using a Bio-Rad GS-690 scanning densitometer (Bio-Rad, Hemel Hempstead, UK) and were normalised by relevant *β*-actin or GAPDH values.

### 2.6. Statistical Methods

For histomorphometry, differences between categorical and continuous variables were analysed by using an independent Student's *t*-test in case of normally distributed data, and a Mann-Whitney *U* test in case of not normally distributed data. Differences between ordinal and continuous data were analysed by using a Kruskal-Wallis test, in case of not normally distributed data. Differences between dichotomous and categorical data were analysed by using a Chi-square test. Differences between 2 continuous variables were analysed with a Spearman correlation test in case of not normally distributed data. In all cases *P* < 0.05 was considered significant. All statistical analysis was performed with SPSS version 17 (SPSS Inc., Chicago, IL, USA). Quantitative RT-PCR results normalised to the values for 18 s RNA were transformed to logarithms and analysed by ANOVA followed by Student's *t*-test with Bonferroni correction.

## 3. Results

### 3.1. MMP-14 and TIMP-3 mRNA Expression in Human Primary Macrophages and FCMs* In Vitro*

**Figure 1:**
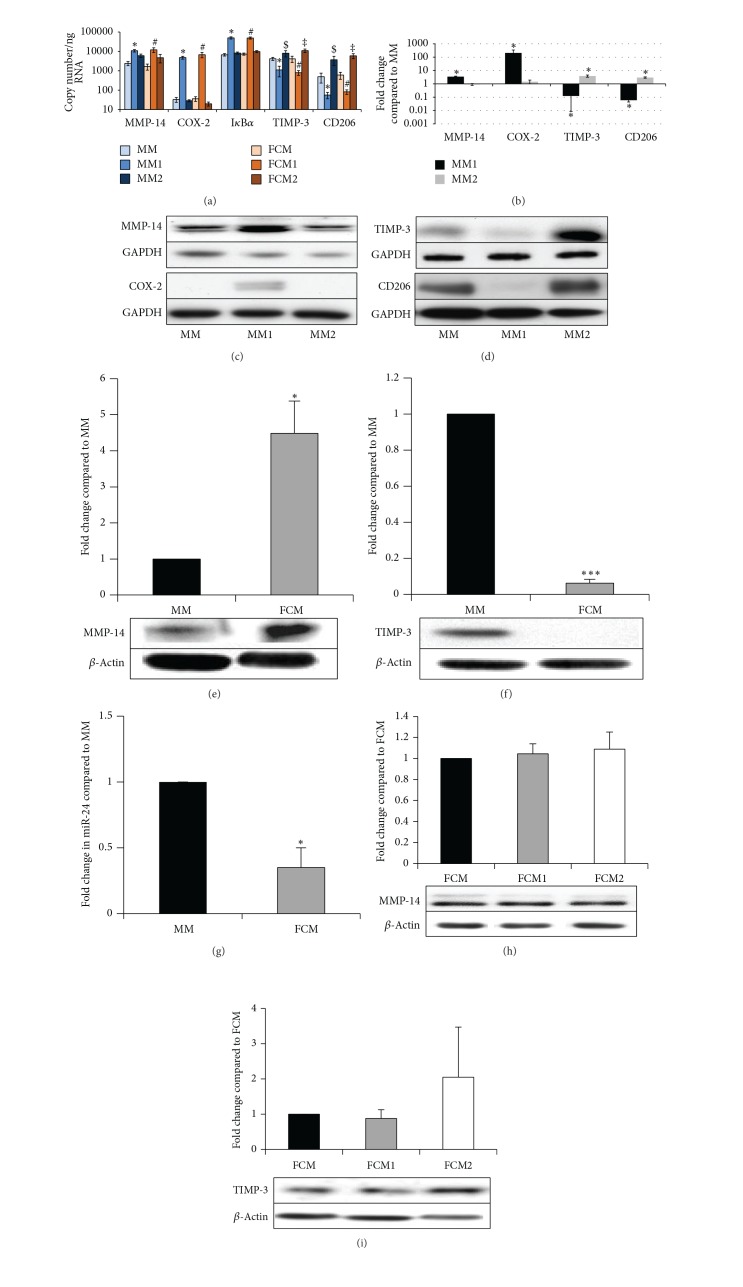
(a) mRNA expression of M-CSF differentiated macrophages (MM) and foam-cell macrophages (FCM) after activation with IFN*γ* and LPS (MM1 and FCM1) or IL-4 (MM2 and FCM2). Data are mean ± SEM; *n* = 7; ∗ = *P* < 0.05 compared to MM and MM2; # = *P* < 0.05 compared to FCM and FCM2; $ = *P* < 0.05 compared to MM and MM1; and ‡ = *P* < 0.05 compared to FCM and FCM1. ((b)–(d)) Densitometric quantification and representative Western blots for MMP-14, COX-2, TIMP-3, and CD206 in M-CSF differentiated macrophages (MM) or after activation with IFN*γ* and LPS (MM1) or IL-4 (MM2). Data are mean ± SEM, *P < 0.05 compared to MM, *n* = 8. ((e)-(f)) Densitometric quantification and representative Western blots for MMP-14 (e) and TIMP-3 (f) in M-CSF differentiated macrophages (MM) and foam-cell macrophages (FCM). Data are mean ± SEM, *P < 0.05 compared to MM, *P < 0.001 compared to MM, *n* = 4. (g) QPCR expression of miR-24 in M-CSF differentiated macrophages (MM) and foam-cell macrophages (FCM). Data are mean ± SEM, *P < 0.05 compared to MM. ((h)-(i)) Densitometric quantification and representative Western blots for MMP-14 (h) and TIMP-3 (i) in foam-cell macrophages (FCM) after activation with IFN*γ* and LPS (FCM1) or IL-4 (FCM2). Data are mean ± SEM; *n* = 4.

Human monocyte-derived macrophages were differentiated with M-CSF (MMs) and some from each batch were treated with oxidised LDL to generate foam-cell macrophages (FCMs), as previously validated in our laboratory [27]. Conversion of MMs to FCMs did not affect mRNA expression of MMP-14 or TIMP-3 (Figure 1(a)) or COX-2 and I*κ*B*α*, markers of proinflammatory activation, or CD206, a gene that is present constitutively but upregulated by anti-inflammatory activation [12, 13] (Figure 1(a)). Proinflammatory activation with LPS and IFN*γ* (referred to as M1 conditions [26]) significantly increased expression of MMP-14 by 4.5- and 7.3-fold, I*κ*B*α* mRNA (a surrogate marker of NF*κ*B signalling [28]) by 7.5- and 6.7-fold, and COX-2 mRNA by 146- and 187-fold, in macrophages (MM1) and FCMs (FCM1), respectively (Figure 1(a)). By contrast, proinflammatory activation decreased mRNA levels of TIMP-3 by 74% and 89% and CD206 by 81% and 86% in macrophages or FCMs, respectively (Figure 1(a)). Anti-inflammatory activation with IL-4 (referred to as M2 conditions [26]) had no effect on MMP-14, COX-2 or I*κ*B*α* mRNA levels but significantly increased TIMP-3 mRNA expression by 1.9- and 2.6-fold and CD206 expression by 7.5- and 9.9-fold in MMs (MM2) and FCMs (FCM2), respectively (Figure 1(a)).

### 3.2. MMP-14 and TIMP-3 Protein Expression in Macrophages and FCMs* In Vitro*

Consistent with the mRNA data, proinflammatory activation significantly elevated protein levels of MMP-14 and COX-2 and significantly decreased those of TIMP-3 and CD206 in nonfoamy macrophages (Figures 1(b) and 1(c)). Anti-inflammatory activation (MM2) also increased macrophage TIMP-3 and CD206 protein levels (Figures 1(b) and 1(d)), consistent with the mRNA data. We conclude that proinflammatory macrophages are MMP-14^hi^  TIMP-3^lo^ whereas unactivated and anti-inflammatory stimulated macrophages are comparatively MMP-14^lo^  TIMP-3^hi^. Foam-cell formation significantly increased protein expression of MMP-14 and decreased TIMP-3 levels (Figures 1(e) and 1(f)), despite not changing the mRNA levels, in agreement with our previous findings in during rabbit macrophage foam-cell formation [7]. These results could be explained by the additional involvement of a posttranscriptional mechanism, such as regulation by microRNA (miR). Indeed, we recently showed that high levels of miR-24 in nonfoamy macrophages limit macrophage MMP-14 protein expression [29]. We observed a 70% decrease in miR-24 levels in FCMs compared to MMs (Figure 1(g)), consistent with the increased MMP-14 protein levels. Interestingly, in contrast to mRNA expression, pro- or anti-inflammatory activation of FCMs did not affect MMP-14 protein expression (Figure 1(h)), which suggests that the effect of the miR is dominant. Pro- or anti-inflammatory activation of FCMs did not affect TIMP-3 protein expression either when compared to nonactivated FCMs (Figure 1(i)). TIMP-3 protein expression may also be subject to miR regulation but this has not yet been demonstrated in macrophages. In summary, our results imply that nonfoamy macrophages are MMP-14^hi^  TIMP-3^lo^ when classically activated whereas FCMs are MMP-14^hi^  TIMP-3^lo^ at the protein level irrespective of their activation status.

### 3.3. Localisation of MMP-14 and TIMP-3 in Human Carotid Atherosclerotic Plaques

**Figure 2:**
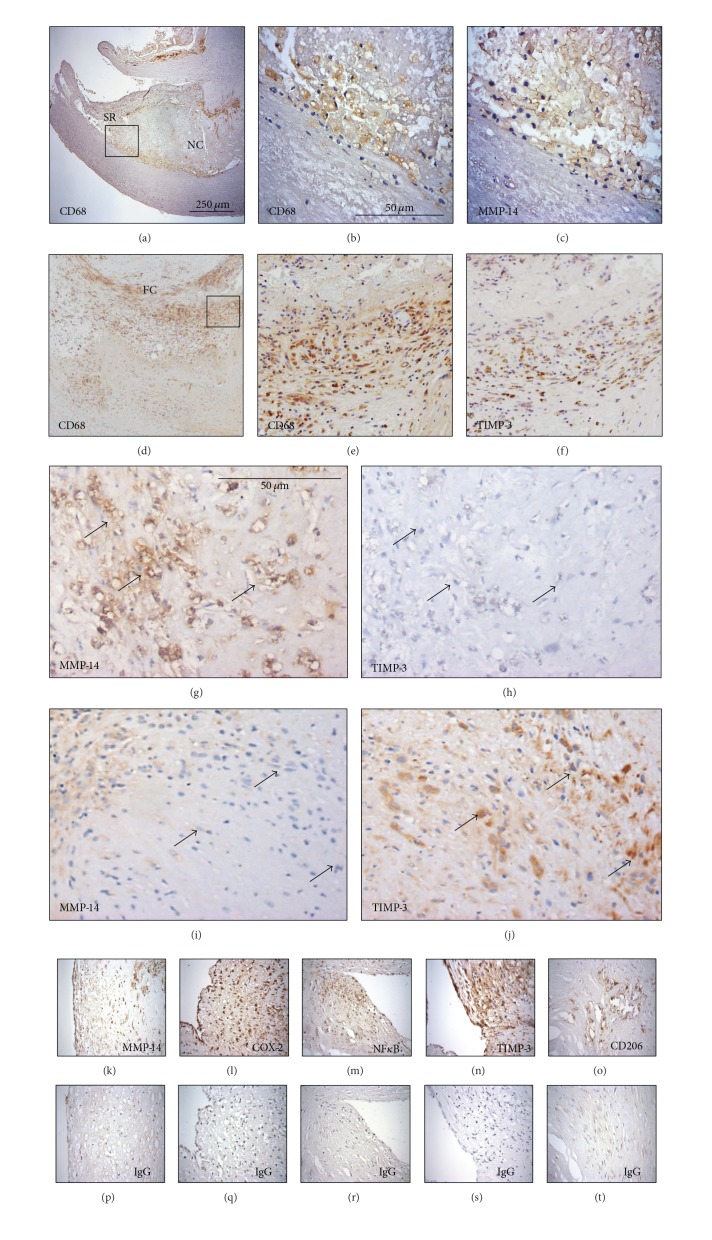
((a)–(f)) Immunolocalisation of macrophages (CD68), MMP-14, and TIMP-3 in human atheromatous ((a)–(c)) and fibrous-atheromatous ((d)-(e)) carotid plaques. Panels (b)-(c) and (e)-(f) are higher magnification fields of boxes depicted in panels (a) and (d), respectively. SR denotes shoulder region, NC denotes necrotic core, and FC denotes fibrous cap. ((g)–(j)) Immunohistochemistry of atheromatous carotid plaques demonstrating MMP-14^hi^  TIMP-3^lo^ ((g) and (h), serial sections) and MMP-14^lo^  TIMP-3^hi^ ((i) and (j), serial sections) foam-cell macrophage regions. Arrows indicate same cells in adjoining serial sections. ((k)–(t)) Immunohistochemical staining (brown product colour) of carotid plaque sections for (k) MMP-14, (l) COX-2, (m) NF*κ*B, (n) TIMP-3, and (o) CD206 and their relevant control IgG on serial sections ((p)–(t), resp.).

**Figure 3:**
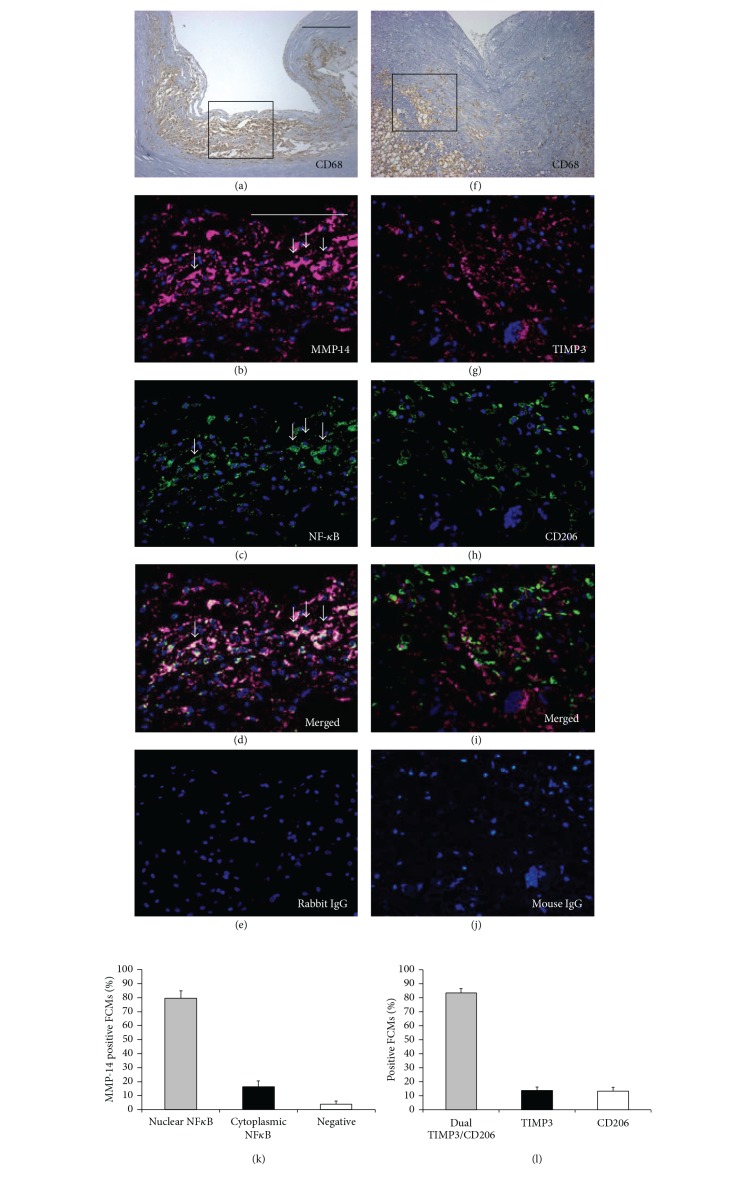
(a)–(j) Representative images of immunohistochemical labelling of unstable ((a)–(e)) and stable ((f)–(j)) human carotid artery atherosclerotic plaques, for macrophages (CD68, panels (a) and (f)), and colocalisation of nuclear-localised NF*κ*B with MMP-14 ((b)–(d)), and CD206 with TIMP-3 ((h)–(j)), with nuclei counterstained with DAPI (blue). Box in panels (a) and (f) represents higher magnification in panels (b)–(e) and (g)–(j), respectively. Scale bar in (a) represents 250 *μ*m and is applicable to panels (a) and (f), whereas scale bar in (b) represents 100 *μ*m and is applicable to panels (b)–(e) and (g)–(j). Arrows in panels (b)–(d) indicate MMP-14 +ve FCMs with nuclear NF*κ*B. Panels (e) and (j) represent negative controls where the primary antibodies were replaced with the relevant species IgG. (k) The percentage of MMP-14 positive FCMs that also stained for nuclear-localised NF*κ*B was significantly greater (***P < 0.001) compared to only cytoplasmic NF*κ*B or no staining. (l) The percentage of TIMP-3 positive FCMs that stained also for CD206 was significantly greater (***P < 0.001) compared to those stained for TIMP-3 or CD206 alone.

**Figure 4:**
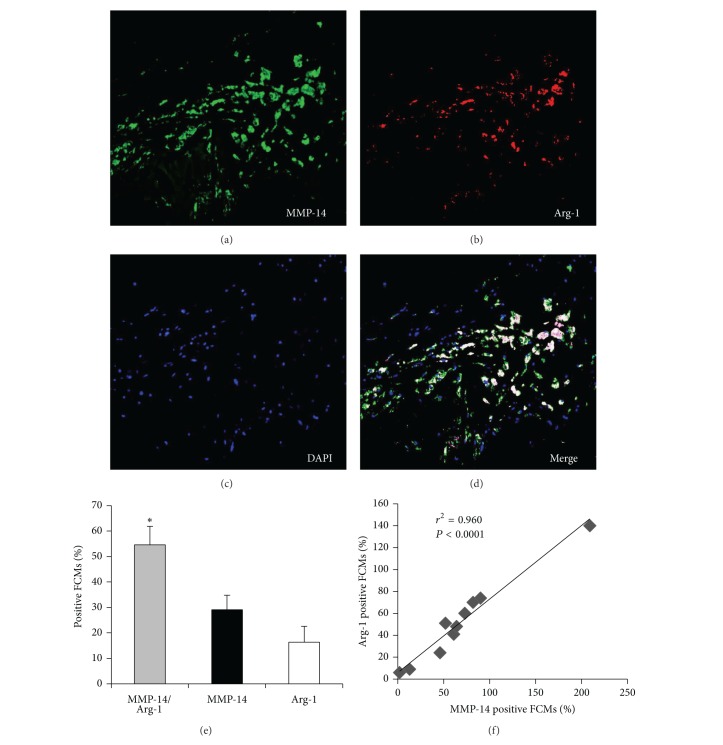
((a)–(d)) Representative images of immunohistochemical labelling of advanced human carotid artery atherosclerotic plaques, for colocalisation of MMP-14 (a) with arginase-1 (b), with nuclei counterstained with DAPI (blue; (c)). Scale bar in (a) represents 100 *μ*m and is applicable to panels (a)–(d). (e) The percentage of MMP-14 positive FCMs that also stained for arginase-1 was significantly greater compared to only MMP-14 or arginase-1 (*P < 0.05; *n* = 10 per group; data expressed as mean ± SEM). (f) Correlation of arginase-1 and MMP-14 positive macrophages in human carotid artery atherosclerotic plaques (*r* ^2^ = 0.960; *P* < 0.0001; *n* = 10).

**Figure 5:**
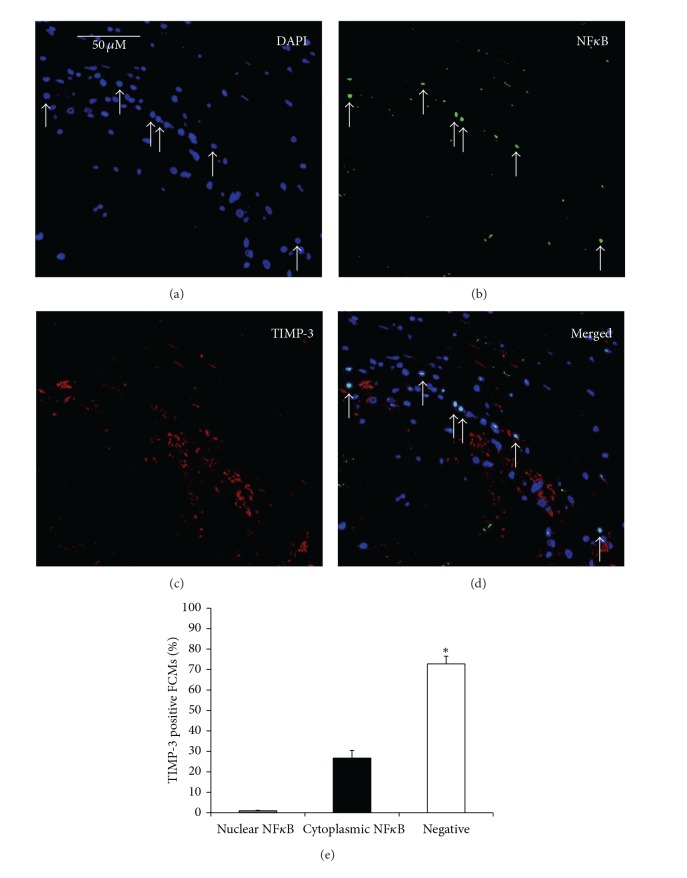
Immunohistochemical staining of carotid plaque sections for (a) 4′,6-diamidino-2-phenylindole (DAPI), (b) NF*κ*B, (c) TIMP-3, and (d) merged, demonstrating that the percentage of TIMP-3 positive FCMs with no nuclear NF*κ*B was significantly greater (*n* = 20, *P < 0.0001) compared to cytoplasmic or nuclear NF*κ*B staining, as depicted in adjoining graph (e).

Macrophages and FCMs were identified in plaques using immunohistochemistry for CD68. The CD68 positive cells varied widely in morphology from large, highly foamy cells to those with very few or no lipid inclusions. We counted all cells irrespective of morphology. MMP-14 positive macrophages/FCMs were found predominantly in the shoulder regions (SR) of atheromatous carotid plaques (Figures 2(a)–2(c)), whereas TIMP-3 positive macrophages/FCMs occurred predominantly within and around the fibrous cap (FC) of fibrous atheromatous plaques (Figures 2(d)-2(e)). The specificity of the antibodies used is demonstrated in Figure 2. In line with our previous findings [7], regions of plaques tended to be either MMP-14 positive and TIMP-3 negative (Figure 2(g) compared to Figure 2(h)) or MMP-14 negative and TIMP-3 positive (Figure 2(i) compared to Figure 2(j)). Dual Immunohistochemistry (as described previously [24]) revealed that in plaque regions with abundant CD68 positive FCMs, MMP-14 colocalised with nuclear-localised NF-*κ*B p65 subunit, a recognised marker of proinflammatory activation. When counted, approximately 80% of MMP-14 positive FCMs also had nuclear-localised NF*κ*B (Figures 3(a)–3(d)), which implies that proinflammatory activation associated with MMP-14 upregulation in macrophages/FCMs in plaques. To gain further insight, we employed arginase-1, which is a marker for anti-inflammatory mouse macrophages [30] and is downregulated in a subpopulation of human FCMs in advanced plaques [31]. Arginase-1 and MMP-14 were coexpressed in more than 50% of intraplaque macrophages/FCMs and the number of arginase-1 and MMP-14 positive macrophages was correlated in advanced plaques (*r* ^2^ = 0.960; *P* < 0.0001, Figure 4). These results are consistent with our* in vitro* data showing high MMP-14 protein levels in FCMs irrespective of M1/M2 activation state (Figure 4). Conversely to MMP-14, less than 1% of TIMP-3 positive macrophages/FCMs demonstrated nuclear-localised NF-*κ*B p65 (Figure 5), implying that few were classically activated. Approximately 80% of macrophages/FCMs that were TIMP-3 positive also stained for CD206, which we showed to be expressed in unactivated (MM) and alternatively activated (MM2) macrophages (Figures 3(f)–3(i)). We therefore concluded that CD206 and TIMP-3 positive macrophages/FCMs in plaques could be unactivated or IL-4 activated.

### 3.4. Relationship of MMP-14 and TIMP-3 Staining to Histological Features of Plaque Stability and Occurrence of Symptoms in Carotid Atherosclerotic Plaques

**Figure 6:**
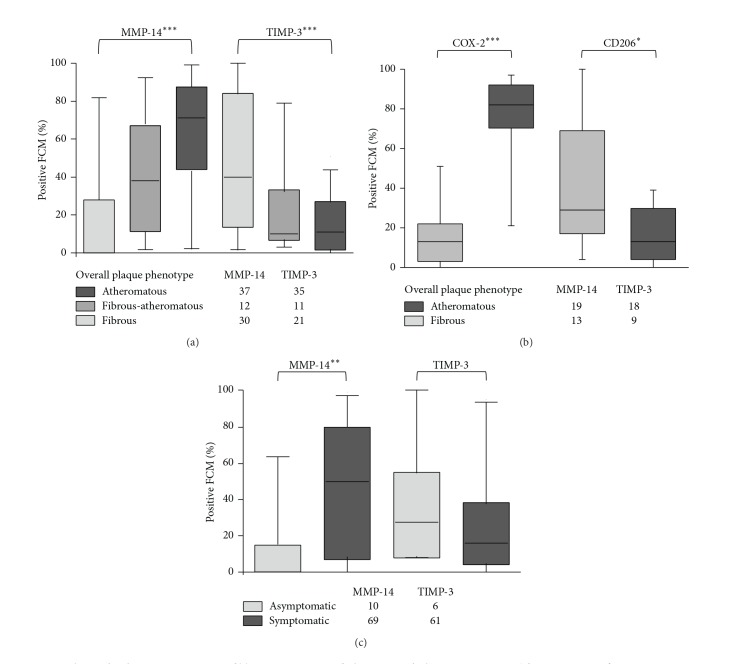
Relationship between MMP-14 and TIMP-3 staining and plaque morphology or symptoms. The percentage of CD68 positive FCMs that stained also for (a) MMP-14 and TIMP-3, or (b) COX-2 and CD206, was related to atheromatous, fibrous-atheromatous and fibrous carotid plaques. (c) Association of FCMs that stained also for MMP-14 and TIMP-3 was assessed in symptomatic and asymptomatic patients. Data analyzed using a Kruskal-Wallis test (*P < 0.05, **P < 0.01, ***P < 0.001). Patient numbers within each group are depicted below each bar graph.

We next investigated the association between MMP-14 and TIMP-3 staining and plaque composition. Carotid plaques entered into AtheroExpress are given an initial classification into atheromatous, fibrous-atheromatous, or fibrous plaques based on a large lipid core, high abundance of macrophages, and low abundance of smooth muscle cells (SMCs) and collagen content [20, 21, 32] (Table 3). Of note, male and older patients are overrepresented in the group with atheromatous plaques (Table 4). By comparison with these previous assignments we found that the percentage of MMP-14 (Figure 6(a)) and COX-2 (Figure 6(b)) positive macrophages/FCMs was significantly greater in atheromatous or fibrous-atheromatous compared to fibrous plaques. By contrast, the percentage TIMP-3 (Figure 6(a)) and CD206 (Figure 6(b)) positive macrophages/FCMs increased in fibrous compared to fibrous-atheromatous and atheromatous carotid plaques. Despite the small number of asymptomatic patients in our cohort, significantly higher MMP-14 positivity was found in plaques from symptomatic compared to asymptomatic patients (Figure 6(c)). There was also a trend towards lower TIMP-3 positivity in plaques from symptomatic patients (Figure 6(c)).

**Figure 7:**
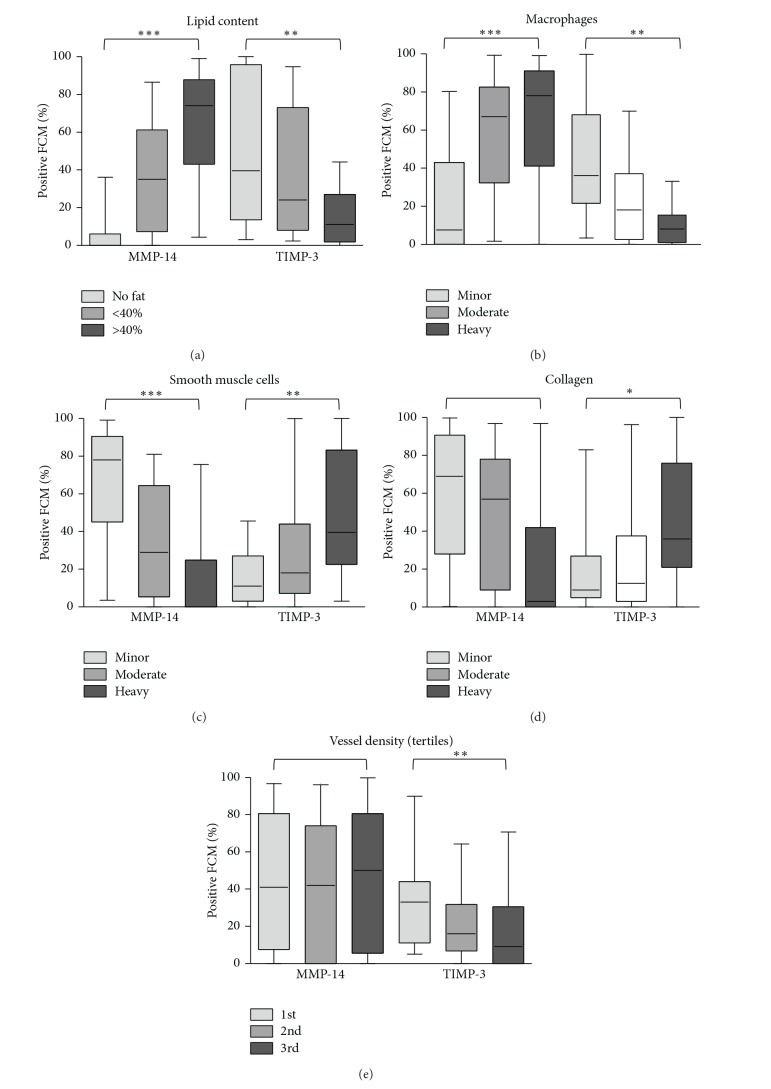
The percentage of MMP-14 and TIMP-3 positive foam-cell macrophages (median [95% confidence limits]) was compared with semiquantitative plaque features and *P* values computed with a Kruskal-Wallis test (*P < 0.05, **P < 0.01, ***P < 0.001).

Amplifying the relationships to overall plaque designation, we found a higher percentage of MMP-14 positive macrophages/FCMs was associated with plaques showing high lipid and macrophage content or decreased SMC number, which are related to plaque instability, whereas the converse was observed for TIMP-3 positive FCMs (Figure 7). TIMP-3 positive macrophages/FCMs were also significantly associated with increased collagen content and reduced plaque neovascularisation (Figure 7), other markers of plaque stability. Furthermore, the percentage of MMP-14 positive macrophages/FCMs across all the sections correlated strongly with total macrophage number and negatively with SMC number (Table 5). Percentage positivity for MMP-14 and COX-2 also correlated with each other (Table 5). Conversely, the percentage of TIMP-3 positive macrophages/FCMs strongly correlated negatively with macrophage number and positively with SMC number (Table 5). Positivity for TIMP-3 correlated directly with CD206 and inversely with MMP-14 (Table 5). Other characteristics including the presence of calcification, intraplaque haemorrhage, and thrombus formation were also recorded. Positivity of MMP-14 or TIMP-3 was unrelated to plaque calcification (data not shown). However, plaques with intraplaque haemorrhage had a significantly higher percentage of MMP-14 positive macrophages/FCMs and tended to have a lower percentage of TIMP-3 positive FCMs (Table 6).

## 4. Discussion

Our main new findings are that macrophages activated with proinflammatory stimuli or foam-cell macrophages (FCMs) irrespective of inflammatory activation are MMP-14^hi^  TIMP-3^lo^ compared to unstimulated (M-CSF differentiated; MM) or anti-inflammatory (IL-4 activated; MM2) macrophages. Furthermore, MMP-14 positive FCMs are more abundant and TIMP-3 positive FCMs are less abundant in plaques with vulnerable rather than stable characteristics.

Multiple macrophage and FCM phenotypes have been previously described [33, 34]. Classical macrophage activation* in vitro* with proinflammatory stimuli is characterised by NF-*κ*B-dependent upregulation of a variety of additional proinflammatory mediators and enzyme systems, including COX-2. By contrast,* in vitro* activation with anti-inflammatory stimuli such as IL-4 or IL-13 mediates a distinct transcriptomic response dependent on STAT-6 phosphorylation [26, 33]. Our results demonstrate a prominent effect of classical activation on MMP-14 mRNA expression in macrophages and FCM [35], consistent with previous work on human monocytes [36] and macrophages [35] demonstrating that NF-*κ*B activation facilitates MMP-14 expression. Formation of foam cells per se did not lead to pro- or anti-inflammatory macrophage activation (Figure 1), consistent with previous studies [16, 37]. However, transformation to FCM increased MMP-14 protein expression, regardless of proinflammatory activation. FCMs appear resistant to M1 and M2 cytokine-stimulation but employ a posttranscriptional pathway to modulate MMP-14 protein expression and subsequent activity. Indeed we have recently identified the involvement of a microRNA, miR-24, in the direct regulation of macrophage MMP-14 protein expression [29]. Further evidence for a novel pathway in regulating FCM MMP-14 protein expression is provided by our demonstration that MMP-14 positive macrophages/FCMs in plaques correlated with classical activation markers, COX-2 and nuclear-localised NF-*κ*B, and with the well-recognised marker of alternative activation, arginase-1. Hence foam-cell formation rather than M1 or M2 macrophage polarisation appears responsible for the MMP-14^hi^ FCMs detected in advanced human atherosclerotic plaques.

By contrast to MMP-14, TIMP-3 mRNA was decreased during proinflammatory activation of macrophages or during foam-cell formation. A combination of these factors probably accounts for the TIMP-3^lo^ populations seen in plaques. TIMP-3 positive macrophages/FCMs correlated and colocalised with the mannose receptor (MR), CD206. In agreement with other previous studies [38], we found that CD206 and TIMP-3 were highly expressed in macrophages differentiated in M-CSF (MM) and upregulated by IL-4 (MM2). However, TIMP-3 expression was only doubled after IL-4 treatment (Figure 1) and is therefore present in both phenotypes. MR positive macrophages/FCMs were previously reported to be mainly located away from the lipid core of plaques [12, 13], consistent with the location of TIMP-3 positive macrophages/FCMs in our study. Although little is known about the regulation of TIMP-3 transcription [39], the striking decrease in protein expression we observed upon foam-cell formation is consistent with previous observations that posttranscriptional mechanisms can regulate TIMP expression during macrophage development [40]. Studies beyond the present scope are going on in our laboratories to investigate this possibility.

Injurious roles for MMP-14 and protective effects of TIMP-3 have been suggested from animal studies (see the Introduction). Consistent with these proposals, we found that MMP-14 positive FCM subpopulations were associated with histological features of plaque vulnerability in carotid plaques based primarily on correlating quantitative plaque characteristics derived from all the sections irrespective of histological appearance (Table 4). The conclusions were also confirmed in carotid atherosclerotic plaques by prior blinded assignment of lesions based on overall histological appearance (Figures 6 and 7). This second analysis can be criticised because it is based on subjective visual observation, although we have shown previously that there is good intraobserver agreement [23]. It is also evident that there is a gender imbalance between the differing plaque phenotypes which may act as a source of variability in the statistical analysis. Nonetheless, collectively our results demonstrate that MMP-14 positive FCMs associate with plaque vulnerability. Whereas TIMP-3 positive macrophages correlate with plaque stability.

In summary our findings show that FCMs in vulnerable atherosclerotic plaques can exhibit increased MMP-14 and decreased TIMP-3 protein expression. This leads to heightened invasive capability, increased proliferation, and augmented susceptibility to apoptosis, as we have previously demonstrated [7]. Our work therefore suggests that reducing MMP-14 activity and increasing that of TIMP-3 could be valid therapeutic approaches to reduce plaque rupture and myocardial infarction.

## Figures and Tables

**Table 1:** Antibodies used for immunohistochemistry.

Antibody	Supplier	Cat. no.	Species	Dilution (Western)	Dilution (IHC)	Secondary antibody
Arginase-1	Santa Cruz	sc-20150	Rabbit	n/a	1/50	Goat anti-rabbit
CD206	R&D	AF2534	Goat	1/100	1/50	Chicken anti-goat
CD68	Dako	M0876	Mouse	n/a	1/100	Chicken anti-mouse
COX-2	Abcam	AB15191	Rabbit	1/2000	1/200	Goat anti-rabbit
MMP-14	Millipore	MAB3317	Mouse	1/1000	1/200	Chicken anti-mouse
NF-kB (p65)	Abcam	AB7970	Rabbit	1/500	1/50	Goat anti-rabbit
TIMP-3	Millipore	MAB3318	Mouse	1/1000	1/200	Chicken anti-mouse

**Table 2:** Primers for quantitative RT-PCR.

Primer		Sequence	Annealing temp. (°C)	Fragment size (bp)
MMP-14	Forward	GGAGACAAGCATTGGGTGTT	60	343
	Reverse	GGTAGCCCGGTTCTACCTTC		
TIMP-3	Forward	CTTCCGAGAGTCTCTGTGGCCTTA	60	230
	Reverse	CTCGTTCTTGGAAGTCACAAAGCA		
COX-2	Forward	GCCATGGGGTGGACTTAAATCATA	60	168
	Reverse	CAGGGACTTGAGGAGGGTAGATCA		
CD206	Forward	CGGTGACCTCACAAGTATCCACAC	58	216
	Reverse	TTCATCACCACACAATCCTCCTGT		
IkB*α*	Forward	CTACTGGACGACCGCCACGACAGC	60	58
	Reverse	CGAGGCGGATCTCCTGCAGCTCCTTG		
36B4	Forward	GCCAGCGAAGCCACGCTGCTGAAC	60	76
	Reverse	CGAACACCTGCTGGATGACCAGCCC		

**Table 3:** Atherosclerotic plaque characteristics.

Plaque characteristics	Plaque phenotype	Gradation				*P* value
% fat/atheroma No/<40%/>40%	Fibrous	15 (50%)		15 (50%)	0 (0%)	0.0001
	Fibrous-atheromatous	0 (0%)		11 (92%)	1 (8%)	
	Atheromatous	0 (0%)		2 (5%)	35 (95%)	

Collagen semiquantitative (minor/moderate/heavy)	Fibrous	1 (4%)		14 (45%)	15 (52%)	0.0001
	Fibrous-atheromatous	2 (17%)		7 (58%)	3 (25%)	
	Atheromatous	17 (46%)		19 (51%)	1 (3%)	

SMC semiquantitative (no/minor/moderate/heavy)	Fibrous	0 (0%)	0 (0%)	10 (33%)	20 (67%)	0.0001
	Fibrous-atheromatous	0 (0%)	3 (25%)	7 (58%)	2 (17%)	
	Atheromatous	2 (5%)	30 (81%)	5 (14%)	0 (0%)	

SMC quantitative % plaque area median [IQR]	Fibrous	4.31 [1.73–5.69]				0.0001
	Fibrous-atheromatous	1.31 [0.39–4.62]				
	Atheromatous	0.53 [0.31–1.07]				

Macrophage semiquantitative (no/minor/moderate/heavy)	Fibrous	7 (23%)	20 (67%)	3 (10%)	0 (0%)	0.0001
	Fibrous-atheromatous	3 (25%)	6 (50%)	2 (17%)	1 (8%)	
	Atheromatous	0 (0%)	0 (0%)	19 (51%)	18 (49%)	

Macrophage quantitative % plaque area median [IQR]	Fibrous	0.20 [0.05–0.63]				0.0001
	Fibrous-atheromatous	0.15 [0.06–0.50]				
	Atheromatous	1.13 [0.60–2.52]				

Thrombus present (no/minor/moderate/heavy)	Fibrous	13 (43%)	14 (47%)	2 (7%)	1 (3%)	0.0001
	Fibrous-atheromatous	2 (17%)	7 (58%)	1 (8%)	2 (17%)	
	Atheromatous	4 (11%)	10 (27%)	16 (43%)	7 (19%)	

CD34 staining Number of vessels median [IQR]	Fibrous	6.7 [3.8–9.5]				0.5000
	Fibrous-atheromatous	6.5 [3.8–10.4]				
	Atheromatous	7.0 [4.3–11.0]				

*P* values for differences among the three groups of semiquantitative histological markers and percentage of macrophages and smooth muscle cells were determined using the Kruskal-Wallis test.

**Table 4:** Patient characteristics.

Patient characteristics	Fibrous plaques (*n* = 30)	Fibrous-atheromatous (*n* = 12)	Atheromatous plaques (*n* = 37)	*P* value
Symptomatic patients *n* (%)	22 (73%)	11 (92%)	36 (97%)	0.012∗
Amaurosis fugax *n* (%)	6 (20%)	5 (42%)	1 (3%)	0.010∗
TIA *n* (%)	12 (40%)	4 (33%)	25 (68%)	
Stroke *n* (%)	4 (13%)	2 (17%)	10 (27%)	
Asymptomatic *n* (%)	8 (27%)	1 (8%)	1 (3%)	
M (♂)/F (♀)	11 ♂ 19 ♀	11 ♂ 1 ♀	34 ♂ 3 ♀	0.007∗
Age mean (sd)	65 (9.6)	73 (8.1)	70 (7.7)	0.016∗
Hypertension *n* (%)	22 (73%)	10 (83%)	31 (84%)	0.540
Smoking *n* (%)	9 (30%)	5 (42%)	13 (35%)	0.761
Diabetes *n* (%)	5 (17%)	0 (0%)	4 (11%)	0.304
Hypercholesterolemia *n* (%)	17 (57%)	6 (50%)	20 (54%)	0.924
Positive family history of heart disease	7 (23%)	2 (17%)	10 (27%)	0.761
History of coronary intervention *n* (%)	7 (23%)	3 (25%)	8 (22%)	0.967
History of peripheral intervention *n* (%)	6 (20%)	1 (8%)	10 (27%)	0.379
History of myocardial infarction	4 (13%)	3 (25%)	1 (3%)	0.064
Statins	16 (53%)	5 (42%)	26 (70%)	0.147
Oral anticoagulants	6 (20%)	4 (33%)	3 (8%)	0.098
Aspirin	18 (60%)	3 (25%)	17 (46%)	0.114
Carbasalate calcium	8 (27%)	8 (67%)	20 (54%)	0.023∗
Aspirin or Carbasalate calcium	24 (80%)	9 (75%)	34 (92%)	0.237
Dipyridamole	18 (60%)	4 (33%)	22 (60%)	0.238
Clopidogrel	3 (10%)	0 (0%)	7 (19%)	0.198
Diuretics	9 (30%)	2 (17%)	11 (30%)	0.881
Beta blockers	13 (43%)	3 (25%)	18 (49%)	0.355
Calcium antagonists	6 (20%)	3 (25%)	12 (32%)	0.514
ACE inhibitors	11 (37%)	3 (25%)	12 (33%)	0.764
Angiotensin II inhibitors	5 (17%)	2 (17%)	4 (11%)	0.755
Insulin	2 (7%)	0 (0%)	0 (0%)	0.187
Oral glucose inhibitors	3 (10%)	0 (0%)	4 (11%)	1.000

*P < 0.05.

**Table 5:** Correlations of immunostains with histological parameters for plaque vulnerability in human carotid plaques.

Plaque characteristic	% of MMP-14^hi^ FCMs			% of TIMP-3^hi^ FCMs		
	*n*	rho	*P* value	*n*	rho	*P* value
Macrophage density	79	**0.453**	<**0.001**	68	−***0.316***	***0.009***
SMC density	76	−***0.466***	<***0.001***	66	**0.283**	**0.021**
% of MMP-14^hi^ FCMs	x	x	x	71	−***0.366***	***0.002***
% of COX-2^+^ FCMs	32	**0.726**	<**0.001**	28	−*0.185 *	*0.345 *
% of CD206^+^ FCMs	27	−*0.013 *	*0.948 *	27	**0.606**	**0.001**

Correlations are shown between the percentage of CD68 positive foam-cell macrophages (FCMs) also positive for MMP-14 and TIMP-3 with quantitative histological markers and percentage of CD68 positive for COX-2 and CD206 using Spearman correlation tests. Negative correlations are shown in italics.

**Table 6:** MMP-14 and TIMP-3 expression and plaque characteristics.

Plaque feature	Absent	*n*	Present	*n*	*P* value
MMP-14 (% of positive foam cell macrophages)					
Intraplaque haemorrhage	24 [0–64]	51	67 [28–81]	28	0.007
Any thrombus or haemorrhage	11 [1–48]	24	59 [6–81]	55	0.026

TIMP-3 (% of positive foam cell macrophages)					
Intraplaque haemorrhage	24 [8–43]	44	11 [5–25]	24	0.095
Any thrombus or haemorrhage	28 [8–77]	22	13 [5–34]	46	0.067

The percentage of MMP-14 and TIMP-3 positive foam cell macrophages (median [95% confidence limits]) was compared with semiquantitative plaque features and *P* values computed with a Kruskal-Wallis test.
